# Fabrication of SiCN(O) Aerogel Composites with Low Thermal Conductivity by Wrapping Mesoporous Aerogel Structures over Mullite Fibers

**DOI:** 10.3390/ma15248811

**Published:** 2022-12-09

**Authors:** Wei Wang, Le Pang, Ming Jiang, Yaping Zhu, Fan Wang, Jingwen Sun, Huimin Qi

**Affiliations:** 1 Key Laboratory of Specially Functional Polymeric Materials and Related Technology of Ministry of Education, School of Materials Science and Engineering, East China University of Science and Technology, Shanghai 200237, China; 2 Laboratory of Aerospace Thermal Control Products Advanced Manufacturing Technology, Shanghai Institute of Space Equipment, Shanghai 201109, China

**Keywords:** SiCN(O) ceramic aerogel, composite, pore structure, thermal conductivity, ambient pressure impregnation, thermal insulation optimization

## Abstract

Silicon-based ceramic aerogels obtained by the polymer pyrolysis route possess excellent thermophysical properties, but their poor mechanical properties limit their broader applicability in thermal insulation materials. Herein, SiCN(O) ceramic aerogels were prepared under the toughening effect of a crosslinker (hexamethylene diisocyanate, HDI), which maintains the structural integrity of the aerogel during the wet gel-to-aerogel conversion. The aerogel maintained a high surface area (88.6 m^2^ g^−1^) and large pore volume (0.21 cm^3^ g^−1^) after pyrolysis. Based on this, mullite-fiber-reinforced SiCN(O) aerogels composites with outstanding thermal insulation properties and better mechanical performance were synthesized via ambient pressure impregnation. Furthermore, the effect of the impregnation concentration on the mechanical and insulation properties of the composites was investigated. The results revealed that the composite prepared with a solution ratio of 95 wt.% exhibited a low density (0.11 g cm^−3^) and a low thermal conductivity (0.035 W m^−1^ K^−1^), indicating an ~30% enhancement in its thermal insulation performance compared to the mullite fiber; the mesoporous aerogel structures wrapped on the mullite fibers inhibited the gas thermal conduction inside the composites.

## 1. Introduction

Aerogel is a novel material with a porous network structure, which filled with gaseous dispersion media in a void. Aerogels have attracted extensive interest in many applications, including high-temperature insulation systems [1,2,3], catalyst supports [4,5,6], medication delivery [7,8] and gas sensors [9,10]. At present, silicon-based ceramic aerogels [11,12,13], SiCN ceramic aerogels, derived from polysilazane precursors via the polymer pyrolysis route, have been developed as one of the most potentially utilized materials in high-temperature thermal insulation applications owing to their excellent properties [14,15], including high temperature stability, oxidation resistance, and chemical durability. However, their poor mechanical properties limit their broader applicability to thermal insulation materials [16,17], requiring enhancement by fibers or other enhancers to implement their application [18,19]. Given the challenge of overcoming the intrinsic fragility of silicon-based ceramic aerogels and the excellent performance of mullite fiber mats [20,21,22], adopting fiber mats as the reinforced phase to fabricate ceramic aerogel composites is more promising [23,24].

Meanwhile, by impregnation the hierarchical porous structures wrapped on mullite fibers provided the composite aerogels with mesoporous structures (pore diameter of <50 nm), which enriched the pore size distribution of the porous structure inside the composite aerogels. Furthermore, in porous materials, the gaseous thermal conductivity occupies the greatest proportion of the total thermal conductivity. Hence, taking measures to reduce the gaseous thermal conductivity is particularly necessary. Based on the Knudsen effect [25], the thermal conductivity of gas in a confined space could be described as follows [26,27,28]:

(1) $$k_{g,\mathrm{CS}}=\frac{k_{g,\mathrm{FS}}}{1+k_{n}}k_{n}=\Lambda_{g,0}/d,$$

where $k_{g,\mathrm{FS}}$, $\Lambda_{g,0}$, and d represent the thermal conductivity of gas in free space, the average free path of gas molecules, and the characteristic size of the pore. Equation (1) shows that, as the average free path of gas molecules increases [29] and the pore diameter of porous thermal insulation materials decrease [30,31], the thermal conductivity characteristics of porous materials decrease. In comparison, decreasing the diameter of pores in porous materials is more promising since increasing the free path of gas molecules by the vacuum process may cause the materials to fail in certain situations [26]. As described, introducing ceramic aerogel porous structures into the fiber mat matrix can potentially reduce the thermal conductivity of the composite.

Herein, we used branch-structured polysilazane as the polymer precursor, hexamethylene diisocyanate (HDI) as the cross-linking agent, and dicumyl peroxide (DCP) as the initiator. The double cross-linking reaction made it possible to form precursor wet gels in dilute solutions. Simultaneously, the toughening effect of the flexible carbon chains in the HDI and the freeze-drying method maintained the structural integrity of the gel during the wet gel-to-aerogel conversion process. Unlike the aerogels prepared using crosslinkers with rigid groups exhibiting a relatively large decrease in specific surface area and pore volume after the process of pyrolysis conversion, aerogels synthesized under the effect of the cross-linking agent with flexible carbon chains showed an increasing trend [15,32,33]. Then, the mullite fiber felt was used as the reinforced phase [18,23,34,35,36], by impregnation polysilazane sol to prepare fiber-reinforced SiCN(O) ceramic aerogel composites through gelling aging, solvent-exchanging, freeze-drying, and pyrolytic ceramization. Mullite fiber felt is an excellent reinforcer, because of its lightweight, high-temperature resistance, and noninflammability characteristics [37]. After compounding with SiCN(O) ceramic aerogels that possess abundant hierarchical porous structures, the mesoporous aerogel structures wrapped on the mullite fibers inhibited the gas thermal conduction inside the composites.

Based on this, the polymer-derived aerogel synthesized by the abovementioned procedures exhibited mesoporous structures with a surface area of 67.5 m^2^ g^−1^ and pore volume (pore diameter < 40.3 nm) of 0.19 cm^3^ g^−1^. After pyrolysis at 1000 °C to convert the polymer-derived aerogels to ceramic aerogels, the surface area and pore volume increased to 88.6 m^2^ g^−1^ and 0.21 cm^3^ g^−1^, respectively, showing an increasing trend in the mesoporous structures. After compounding with SiCN(O) ceramic aerogels that possess abundant hierarchical porous structures, the composite aerogels exhibit low density, certain flexibility, and excellent mechanical properties. Additionally, further improvement in the thermal insulation capacity due to the porous aerogel structures encapsulated on the mullite fibers limit the heat transfer inside the SiCN(O) ceramic aerogel composites. Notably, compounding aerogels with fibers improves the mechanical properties of the aerogel materials, changes the internal pore structure of the matrix material, and improves the thermal insulation performance, resulting in ~30% enhancement in the thermal insulation performance compared to the thermal conductivity of mullite fiber felt (0.05 W m^−1^ K^−1^).

## 2. Experimental

### 2.1. Preparation of SiCN(O) Ceramic Aerogel

The liquid-phase branch-structured polysilazane (PSZ-BS, laboratory-made) was used as the polymer precursor. Hexamethylene diisocyanate (HDI, Aladdin Industrial Corporation, Shanghai, China) and dicumyl peroxide (DCP, Sinpharm Chemical Reagent Co., Ltd., Shanghai, China) were used as the cross-linking agent and initiator, respectively. In a typical synthesis procedure, 0.4 g HDI, 0.6 g PSZ-BS, 19 g toluene (95 wt.% solution ratio), and 0.05 g DCP (5 wt.% of PSZ-BS precursor) were loaded into a Teflon-lined autoclave to form an ultra-diluted solution with stirring for 10 min. The pressure vessel was introduced in a preheated oven at 80 °C and 150 °C for 5 and 20 h, respectively, to obtain the preceramic wet gels. After cooling down to room temperature, the wet gels were removed from the Teflon-lined autoclave and transferred into a beaker filled with toluene to remove the crosslinker and unreacted precursors. The wet gels were washed daily for three days by replacing the toluene solvent with tert-butanol solvent. The polymer-derived aerogels were obtained after freeze-drying at −60 °C for 24 h and were named PSZ-BSAGs. Furthermore, the PSZ-BSAGs were pyrolyzed at 1000 °C to obtain SiCN(O) ceramic aerogel CPSZ-BSAGs. After this, SiCN(O) ceramic aerogels of CPSZ-BSAG-5%, CPSZ-BSAG-10%, and CPSZ-BSAG-15% were obtained using solution ratios of 5, 10, and 15 wt.%, respectively.

### 2.2. Preparation of SiCN(O) Ceramic Aerogel Composite

**Figure 1:**
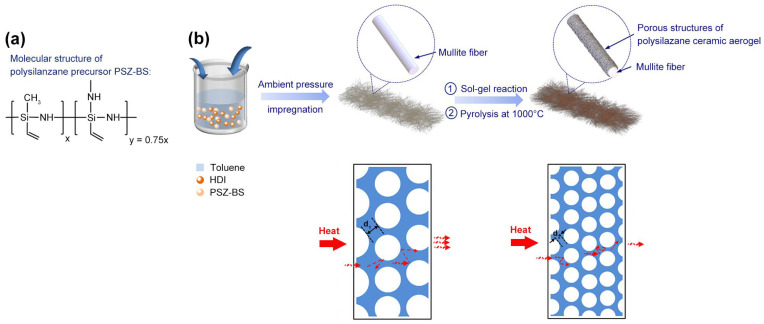
(**a**) Molecular structure of polysilazane precursor PSZ-BS, (**b**) schematic description of the preparation route of ceramic aerogel composite with low thermal conductivity based on reducing the pore size (d_2_ < d_1_) of the composite by compounding with the aerogel structure.

Based on the synthesis of the polymer-derived aerogels (PSZ-BSAGs), a PSZ-BS mixture solution was prepared to impregnate the mullite fiber felt (Shandong Luyang Energy-Saving Materials Co., Ltd., Shanghai, China), and the preparation route of ceramic aerogel composite is illustrated in Figure 1b. Subsequently, the pressure vessel was transferred to a preheated oven and heated at 80 °C for 5 h. Later, the obtained gel composite was aged for 20 h at 150 °C. Then, the wet gel composite was washed daily for three days by replacing the toluene solvent with tert-butanol solvent. Finally, the ceramic aerogel composite was prepared by the freeze-drying method and pyrolyzed at 1000 °C for 2 h. Different ceramic aerogel composites, named M-CPSZ-BSAG-5%, M-CPSZ-BSAG-10%, and M-CPSZ-BSAG-15%, were prepared using 5, 10, and 15 wt.% of the toluene solvent in the precursor mixture solution, respectively.

### 2.3. Characterization

Fourier-transform infrared spectroscopy (FT-IR) data were acquired on a Nicolet 6700 FT-IR spectrometer (Thermo Fisher Scientific Corp., Waltham, MA, USA) at room temperature in the wavenumber region 4000–400 cm^−1^. Nitrogen sorption isotherms were recorded at 77 K using an ASAP2460 Micromeritics apparatus (Micromeritics, Atlanta, GA, USA). Each sample was degassed for several hours at 50 °C under a secondary vacuum. The specific surface area (SSA), pore volume, pore diameter and pore size distribution are determined from the N_2_ adsorption-desorption measurements. The SSAs and pore-size distributions were determined using the Brunauer-Emmett-Teller (BET) method and the Barrett-Joyner-Halenda (BJH) method, respectively. The thermogravimetric and differential thermogravimetric curves of SiCN(O) samples were recorded by a STA 409 thermogravi-metric analyzers (Netzsch, Bavarian, Germany). The analysis was performed up to 1000 °C with a heating rate of 10 °C/min in flowing N_2_. In this way, it was possible to assess the ceramic yield and pyrolytic ceramization process of polysilazane-derived SiCN(O). The morphological features of the SiCN(O) ceramic aerogel composites were analyzed from surface morphologies using a Field Emission Scanning Electron Microscope (FE-SEM) (Hitachi S-4800, Tokyo, Japan) after gold film deposition by sputtering. The thermal conductivity of the SiCN(O) ceramic aerogel composites was acquired on a thermocline-method (i.e., hot-wire method) thermal conductivity analyzers (XIATECH TC3200, Xian, China), the hot-wire method is a transient technique that measures temperature rise at a known distance from a linear heat source embedded in the test sample, which is commonly used to measure low thermal conductivity materials such as powdered materials and porous materials. During the test, the test samples were cut into a cuboid shape (length is 40 nm, width is 30 nm, height is 5 nm), the experimental voltage and test acquisition were 0.8 V and 5 s, respectively, the thermal conductivity test of samples with the same composition were repeated 10 times. The stress-strain curves and the compression test were measured by the Zwick/Roell Z020 machine at room temperature. Samples of composites impregnated with three different precursor concentrations were all cut into a cylinder shape (height is 15 mm, the diameter is 25 mm) before characterization, and the direction of the test is the axial direction of the cylindrical sample.

## 3. Results and Discussion

**Figure 2:**
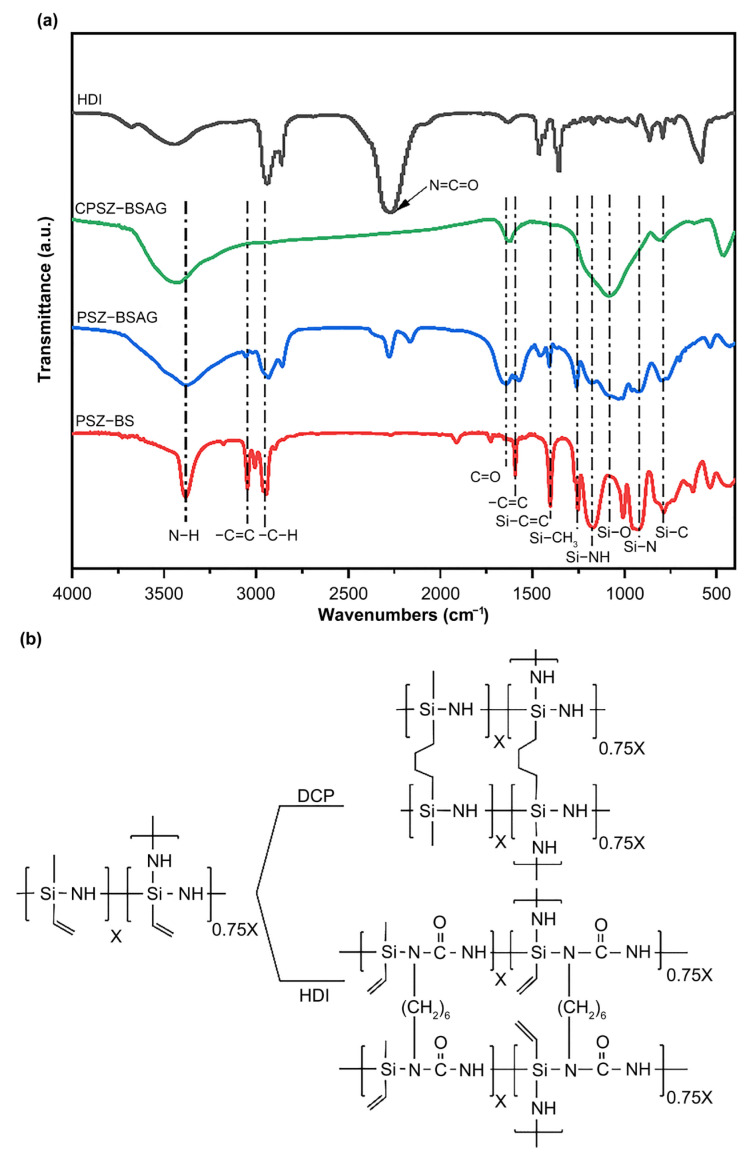
(**a**) FT−IR spectra of reactants (PSZ-BS and HDI), polymer-derived aerogel (PSZ-BSAG) and ceramic aerogel (CPSZ-BSAG). (**b**) Possible reactions during the process of sol–gel reaction.

The Fourier transform infrared (FT−IR) spectra of PSZ-BS, PSZ-BSAG, CPSZ-BSAG, and HDI are shown in Figure 2a. First, typical cross-linking reactions can be seen clearly by comparing the spectra of the reactants (HDI, PSZ-BS, and PSZ-BSAG). The FT-IR spectrum of PSZ-BSAG shows all the functional groups stemming from the reactants. The stretching vibration and deformation bands at 3384 and 1173 cm^−1^ are attributed to the N–H group. The symmetric deformation at ~1255 cm^−1^ (Si–CH_3_ stretching) and the asymmetric stretching band at 2944 cm^−1^ (C–H stretching in Si–CH_3_) indicate the existence of Si–CH_3_. The bands attributed to vinyl silyl groups (CH_2_=CH–Si) are represented by the C–H and C=C stretching vibrations at ~3047 cm^−1^ and 1594 cm^−1^, respectively. The disappearance of most CH_2_=CH– groups at 3047 and 1594 cm^−1^, compared to the PSZ-BS spectrum, can be attributed to the cross-linking reaction of the C=C bonds under the function of the initiator (DCP). The backbone vibrations of Si–N (Si–N–Si) are located at ~940 cm^−1^. The FT-IR spectrum also shows the Si–C deformation at 785 cm^−1^. Additionally, the new peaks at ~2270 cm^−1^ and 1640 cm^−1^, corresponding to the N=C=O and C=O bonds, are due to the reactions between the Si–N bonds of the polymer precursor and the N=C=O bonds of the crosslinker (HDI). The corresponding reactions are shown in Figure 2b. According to the FT−IR spectrum, under the effect of the initiator (DCP), the self-crosslinking reaction occurred among the C=C bonds of the polysilazane precursor in the soluble mixed solution. Meanwhile, the crosslinking reaction between highly reactive groups of crosslinker and precursor also made it possible to form a three-dimensional gel network in dilute solutions (95 wt.% solvent ratio), which could generate more pores after solvent evaporation during the process of freeze-drying. While a possible reason for the incomplete consumption of the N=C=O bonds could be the steric hindrance between reactants. The polymer-to-ceramic transformation can be seen clearly by comparing the spectra of PSZ-BSAG and CPSZ-BSAG. After pyrolysis at 1000 °C, PSZ-BSAG completed the transformation from polymer to ceramics with the disappearance of the C–H and N–H bonds. The Si–O bond can be observed at 1080 cm^−1^ after pyrolysis, which could be attributed to the atomic rearrangement at high temperatures. By this method, the polysilazane organic aerogel completed the transition to inorganic ceramic aerogels, and the SiCN(O) ceramic aerogel was composed of S–N, Si–O, C–N, C–C, C=C, C–O, and C–N bonds.

**Figure 3:**
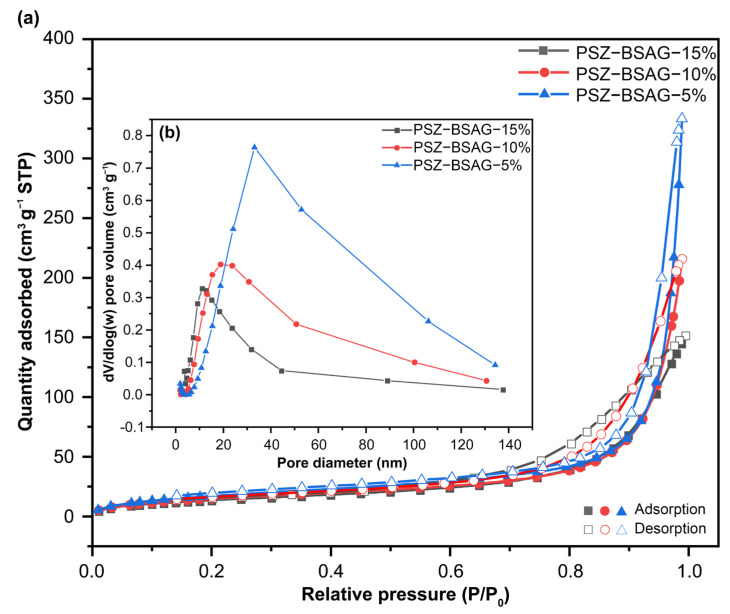
(**a**) Nitrogen adsorption–desorption isotherm and (**b**) pore size distribution of polymer-derived aerogels (PSZ-BSAGs) prepared with different solution ratios.

The changes in the surface area and pore size distribution of the PSZ-BSAGs materials synthesized with different solvent ratios during the sol–gel reactions were characterized by the nitrogen adsorption–desorption experiments (Brunauer–Emmett–Teller (BET) method). The adsorption–desorption isotherms of different samples are shown in Figure 3a. The results indicate that the PSZ-BSAGs are mesoporous materials according to the IUPAC classification. The hysteresis loops could be further classified as H3, where the isotherm does not terminate at high pressure, indicating a considerable fraction of large mesopores and macropores. The BET surface areas of the PSZ-BSAGs are 52.7, 56.8, and 67.5 m^2^ g^−1^, respectively, which increase as the solvent ratio increases (85, 90, and 95 wt.%, respectively); the pore volumes are 0.17, 0.18, 0.19 cm^3^ g^−1^, respectively (Table 1). Moreover, this trend indicates that the volume of the adsorbed gas increases with the solvent ratio, which can be attributed to the fact that more toluene solvent occupies more space during the sol–gel reaction and forms wider pores after freeze-drying. Correspondingly, the pore size distribution changes toward larger sizes with the addition of the solvent (Figure 3b).

**Figure 4:**
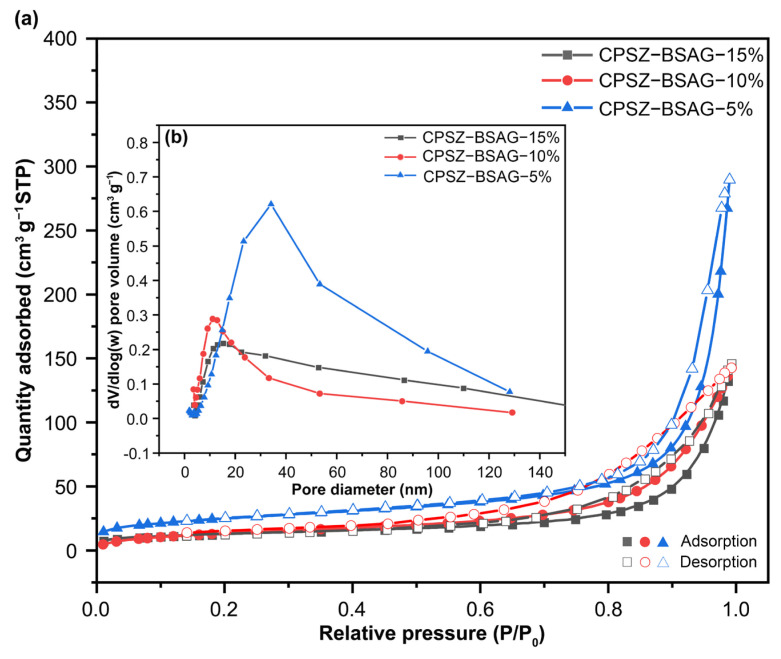
(**a**) Nitrogen adsorption–desorption isotherm and (**b**) pore size distribution of ceramic aerogels (pyrolysis at 1000 °C) prepared with different solution ratios.

After pyrolysis at 1000 °C, the PSZ-BSAG completed the polymer-to-ceramic transformation, and the adsorption–desorption isotherms as well as pore size distribution of ceramic aerogels are shown in Figure 4. The BET surface area and pore volume exhibit varying reductions under the solvent ratios of 85 and 90 wt.% due to the shrinkage effect during pyrolysis (Table 2). Surprisingly, the CPSZ-BSAG-5% (solvent ratio of 95 wt.%) exhibits the phenomenon of an increase in the BET surface area (from 67.5 to 88.6 m^2^ g^−1^) and pore volume (from 0.19 to 0.21 cm^3^ g^−1^). The broadened pore size due to the occupation of more volume by the increase in the solvent during the sol–gel reaction may account for this phenomenon, which would produce more mesopores by transforming more macropores into mesopores during the shrinkage process of the pyrolysis. Differently, the SiCN(O) synthesized using crosslinker with rigid groups, for example divinylbenzene, showed ~65% decrease in specific surface area and pore volume [32,33] after the conversion to ceramic aerogels. While using the crosslinker with flexible carbon chains, the formed three-dimensional gel network possessed relatively lower internal stress, producing more mesopores owing to the shrinkage at high temperatures.

**Figure 5:**
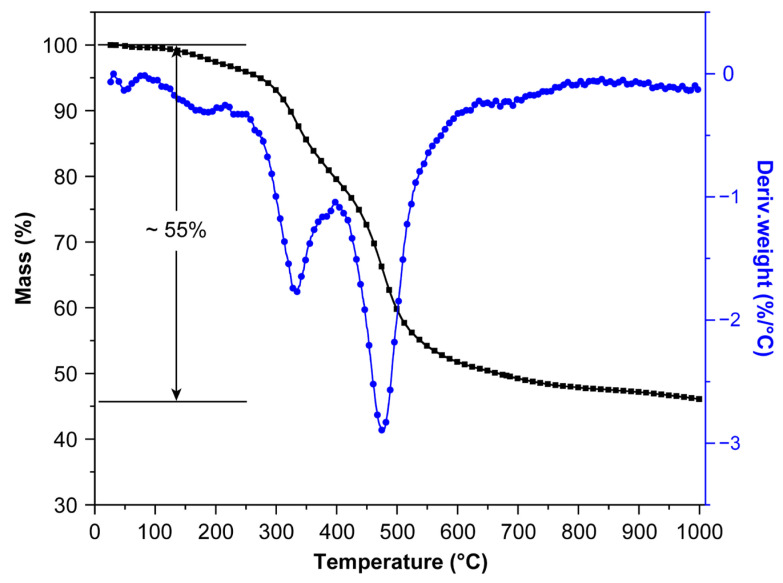
Thermogravimetric (TG)–differential thermogravimetric (DTG) analysis of polymer-derived aerogel (PSZ-BSAG) under nitrogen flow up to 1000 °C.

The polymer-to-ceramic conversion of the PSZ-BSAGs was characterized by thermogravimetric (TG) and differential thermogravimetric (DTG) analysis under a nitrogen atmosphere from 25 °C to 1000 °C (Figure 5). According to the TG curve, the final ceramic yield of the PSZ-BSAG is ca. 45% at 1000 °C, and a major weight loss could be observed at a temperature range of 200–600 °C. Meanwhile, during pyrolysis, the major weight loss occurred in three temperature stages, as shown in the DTG curve. In the first stage (<200 °C), the mass loss is mainly caused by the escape of micromolecules and solvents at high temperatures. In the second stage (20–400 °C), the weight loss is majorly due to the transamination reaction effect. Finally, in the third stage (400–600 °C), the maximum weight loss is experienced, which is primarily attributed to the inorganic transformation under the effect of the molecular chain rearrangement and the cleavage and removal of organic chain segments during the pyrolytic ceramization process of the precursor aerogels. After 600 °C, the mass of the aerogels was almost unchanged; thus, the process of ceramization was basically complete.

**Figure 6:**
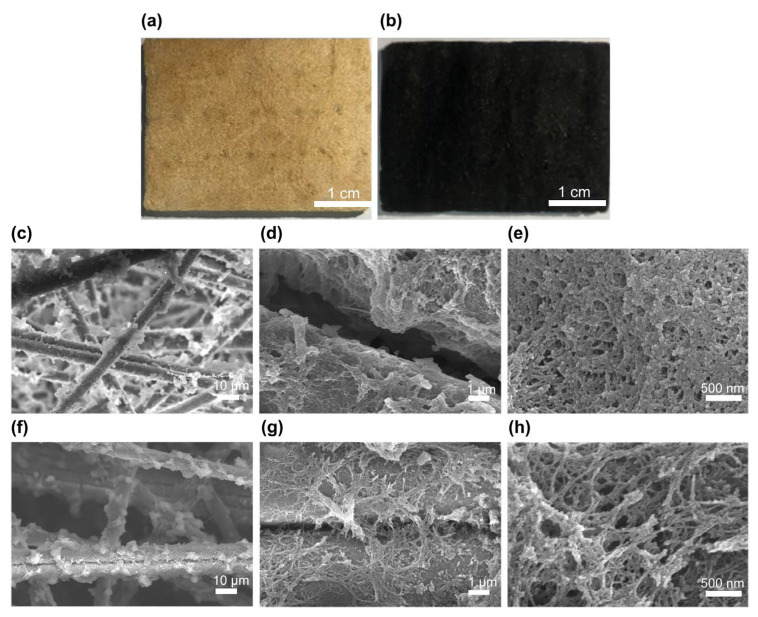
Digital images (**a**) before and (**b**) after pyrolysis and typical scanning electron microscopy (SEM) micrographs of ceramic aerogel composite (**c**–**e**) before and (**g**–**h**) after pyrolysis at 1000 °C.

Based on the solvent ratio effect on the surface area and pore volume of SiCN(O) ceramic aerogels, the ceramic aerogel possesses the largest mesoporous structures under a solvent ratio of 95 wt.%. Therefore, the ceramic aerogel composite was prepared by impregnating the mullite fiber felt with PSZ-BS sol (95 wt.% solvent ratio). The images of the aerogel composite before and after pyrolysis are shown in Figure 6a,b. The color change of the sample is mainly due to the polymer–ceramic transition of the PSZ-BSAG encapsulated on the mullite fiber after pyrolysis. Furthermore, the scanning electron microscopy (SEM) images of the aerogel composite are depicted in Figure 6c–h. The aerogel composite comprises polysilazane-derived aerogel composites (Figure 6c–e) and SiCN(O) ceramic aerogel composites pyrolyzed at 1000 °C (Figure 6f–h). The aerogel structures wrapped on the mullite fibers exhibit a three-dimensional network structure with clusters comprising compact submicron particles. The mullite fibers are important for skeleton support, reinforcing the composite’s backbone. Additionally, the polysilazane-derived aerogel and SiCN(O) ceramic aerogel particles are closely attached to the mullite fibers, indicating good compatibility between the aerogel matrix and the mullite fibers. Finally, according to the Knudsen effect the gaseous thermal conductivity takes the greatest proportion of the total thermal conductivity of porous aerogel materials, and decreasing the pore size is an effective way to reduce the gaseous thermal conductivity. The hierarchical porous structures wrapped on the mullite fibers provide the composite aerogels with mesoporous structures, which change the pore size distribution of aerogel composites, limiting the thermal gas conduction. Meanwhile, the pore diameter clearly decreases, which is due to the volume shrinkage during pyrolysis, corresponding to the characterization results of the nitrogen adsorption–desorption experiments.

**Figure 7:**
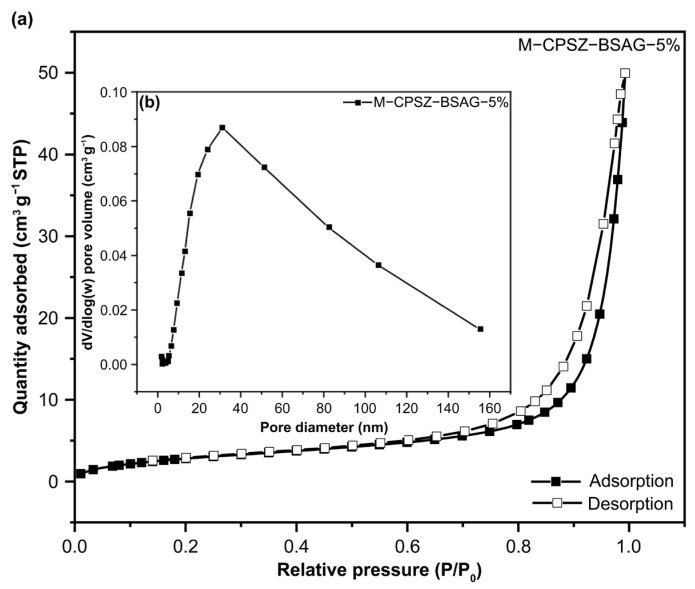
(**a**) Nitrogen adsorption–desorption isotherm and (**b**) pore size distribution of the ceramic aerogel composite (pyrolysis at 1000 °C).

To verify the introduction of the mesoporous structures to the mullite fiber felt by impregnation, nitrogen adsorption–desorption experiments (the BET method) were conducted to characterize the pore structure of the M-CPSZ-BSAG-5% ceramic aerogel. While the weight of the mullite fiber felt matrix accounts for the majority of the composite mass, which may decrease the BET surface area and pore volume to some extent, the pore structure data still exhibit mesopores with a BET surface area of 11.0 m^2^ g^−1^ and a pore volume of 0.03 cm^3^ g^−1^ (Table 3). Additionally, the pore size distribution of M-CPSZ-BSAG-5% reveals an approximate distribution range of 10–160 nm compared with CPSZ-BSAG-5% (Figure 7a). The results suggest that the mullite fiber felt compounded with ceramic aerogels could enrich the pore size distribution of the porous structure inside the composite aerogels, which provides the possibility of reducing the thermal conductivity of the composite. Combined with scanning electron microscopy micrographs of aerogel composites, both characterization methods revealed that by impregnating polysilazane sol the aerogel composites enriched pore structures with smaller pore size compared with mullite fiber matrix, which provides a basis for limiting heat transfer in the gas phase and reducing the thermal conductivity of the porous aerogel material.

**Figure 8:**
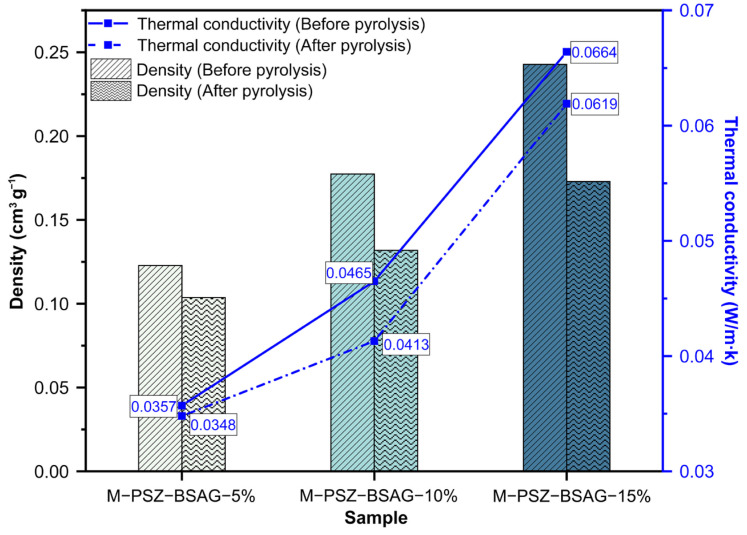
Density and thermal conductivity of aerogel composites prepared with different solution ratios before and after pyrolysis at 1000 °C.

**Figure 9:**
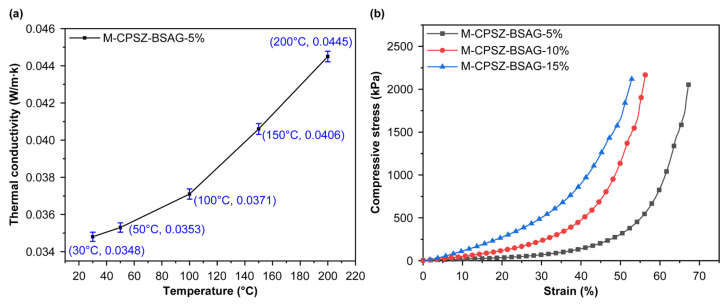
(**a**) Thermal conductivity of M-PSZ-BSAG-5% at different environmental temperatures, and (**b**) compressive stress–strain curves of composites impregnated with different precursor concentrations.

Figure 8 illustrates the densities and thermal conductivities of the aerogel composites prepared with different solvent ratios before and after pyrolysis. Both the thermal conductivities of the aerogel and ceramic aerogel composites increase as the solvent ratios decrease due to the decrease in porosity as the solute impregnation concentration increases, which can be revealed from the increase in density. Meanwhile, the densities and thermal conductivities exhibit different reduction degrees after pyrolysis, which benefit from mass loss, volume shrinkage, and consequent increase in porosity during pyrolysis, corresponding to changes in the microscopic appearance (Figure 6a–c). Especially, the M-CPSZ-BSAG-5% material exhibits the lowest density of ~0.11 g cm^−3^ and thermal conductivity of 0.035 W m^−1^ K^−1^, resulting in ~30% enhancement in its thermal insulation performance compared with the thermal conductivity of the mullite fiber felt (0.050 W m^−1^ K^−1^). To further investigate the thermal insulation performance characteristics of the ceramic aerogels at different ambient temperatures, the thermal conductivity was characterized using a thermal conductivity meter with the hotline method in a temperature range of 30–200 °C (Figure 9a). The high porosity characteristics of ceramic aerogel composites make the proportion of the gas-phase heat conduction significant. As the environmental temperature increases, the thermal movement of gas molecules intensifies, making the gas-phase thermal convection in the pores gradually obvious. The thermal conductivity of the M-CPSZ-BSAG-5% ceramic aerogel composite is 0.0445 W m^−1^ K^−1^ at 200 °C. In addition, Figure 9b illustrates the compressive stress–strain curves of the composites impregnated with different precursor concentrations. The results reveal that the deformation capacity of the composites increases as the precursor concentration decreases. Unlike the fragile characteristics of SiCN(O) ceramic aerogel materials, adopting a mullite fiber mat as a matrix material enhances the deformation properties of the ceramic aerogel composites, which broadens their application in the field of insulation materials.

## 4. Conclusions

Herein, we used branch-structured polysilazane as the polymer precursor, hexamethylene diisocyanate (HDI) as the cross-linking agent, and dicumyl peroxide (DCP) as the initiator to prepare polysilazane-derived aerogels and SiCN(O) ceramic aerogels with structural integrity. Afterward, the effects of the solvent ratio during the sol–gel reaction and pyrolysis process on the Brunauer–Emmett–Teller (BET) surface area and pore volume were studied. Meanwhile, the CPSZ-BS-5% ceramic aerogels exhibited a larger BET surface area of 88.6 m^2^ g^−1^ and pore volume of 0.21 cm^3^ g^−1^, indicating an increasing trend after pyrolysis, which could preserve more pore structures with a small size after pyrolysis by using the cross-linking agent with flexible carbon long chains. Based on these, SiCN(O) ceramic aerogel composites were prepared by impregnating sol, sol–gel reaction, and pyrolysis. Meanwhile, the thermal conductivity of the composites increases as the solvent ratio of the impregnated sol decreases. The optimum solvent ratio was found to be 95 wt.%, at which the SiCN(O) ceramic aerogel composite exhibited a low density of ~0.11 g cm^−3^ and thermal conductivity of 0.035 W m^−1^ K^−1^, showing ~30% enhancement in its thermal insulation performance compared to the mullite fiber felt (0.050 W m^−1^ K^−1^), the mesoporous aerogel structures wrapped on the mullite fibers inhibited the gas thermal conduction inside the composites.

## Figures and Tables

**Table 1:** Pore structure data (characterized by the Brunauer–Emmett–Teller (BET) method) of polymer-derived aerogels (PSZ-BSAGs).

Sample	BET Surface Area (m^2^ g^−1^)	Pore Diameter ^a^ (nm)	Pore Volume ^b^ (cm^3^ g^−1^)
PSZ-BSAG-15%	52.7	12.9	0.17
PSZ-BSAG-10%	56.8	19.8	0.18
PSZ-BSAG-5%	67.5	28.1	0.19

^a^ Pore diameter: average pore diameter determined by the Barrett−Joyner−Halenda theory. ^b^ Pore volume: total pore volume of pores diameter <403 Å.

**Table 2:** Pore structure data (characterized by the BET method) of ceramic aerogels (pyrolyzed at 1000 °C).

Sample	BET Surface Area (m^2^ g^−1^)	Pore Diameter ^a^ (nm)	Pore Volume ^b^ (cm^3^ g^−1^)
CPSZ-BSAG-15%	45.4	17.8	0.13
CPSZ-BSAG-10%	51.2	12.6	0.16
CPSZ-BSAG-5%	88.6	23.2	0.21

^a^ Pore diameter: average pore diameter determined by the Barrett−Joyner−Halenda theory. ^b^ Pore volume: total pore volume of pores diameter <403 Å.

**Table 3:** Pore structure data (characterized by the BET method) of the ceramic aerogel composite (pyrolyzed at 1000 °C).

Sample	BET Surface Area (m^2^ g^−1^)	Pore Diameter ^a^ (nm)	Pore Volume ^b^ (cm^3^ g^−1^)
M-CPSZ-BSAG-5%	11.0	25.1	0.03

^a^ Pore diameter: average pore diameter determined by the Barrett−Joyner−Halenda theory. ^b^ Pore volume: total pore volume of pores diameter <403 Å.

## Data Availability

The data that support the findings of this study are available from the corresponding author upon reasonable request.
